# Supplementary figures and images for: The risk of dyslipidemia on PLHIV associated with different antiretroviral regimens in Huzhou
Source: PLoS One. 2024 Sep 20;19(9):e0305461. doi: 10.1371/journal.pone.0305461 (PMC11414983; doi:10.1371/journal.pone.0305461)

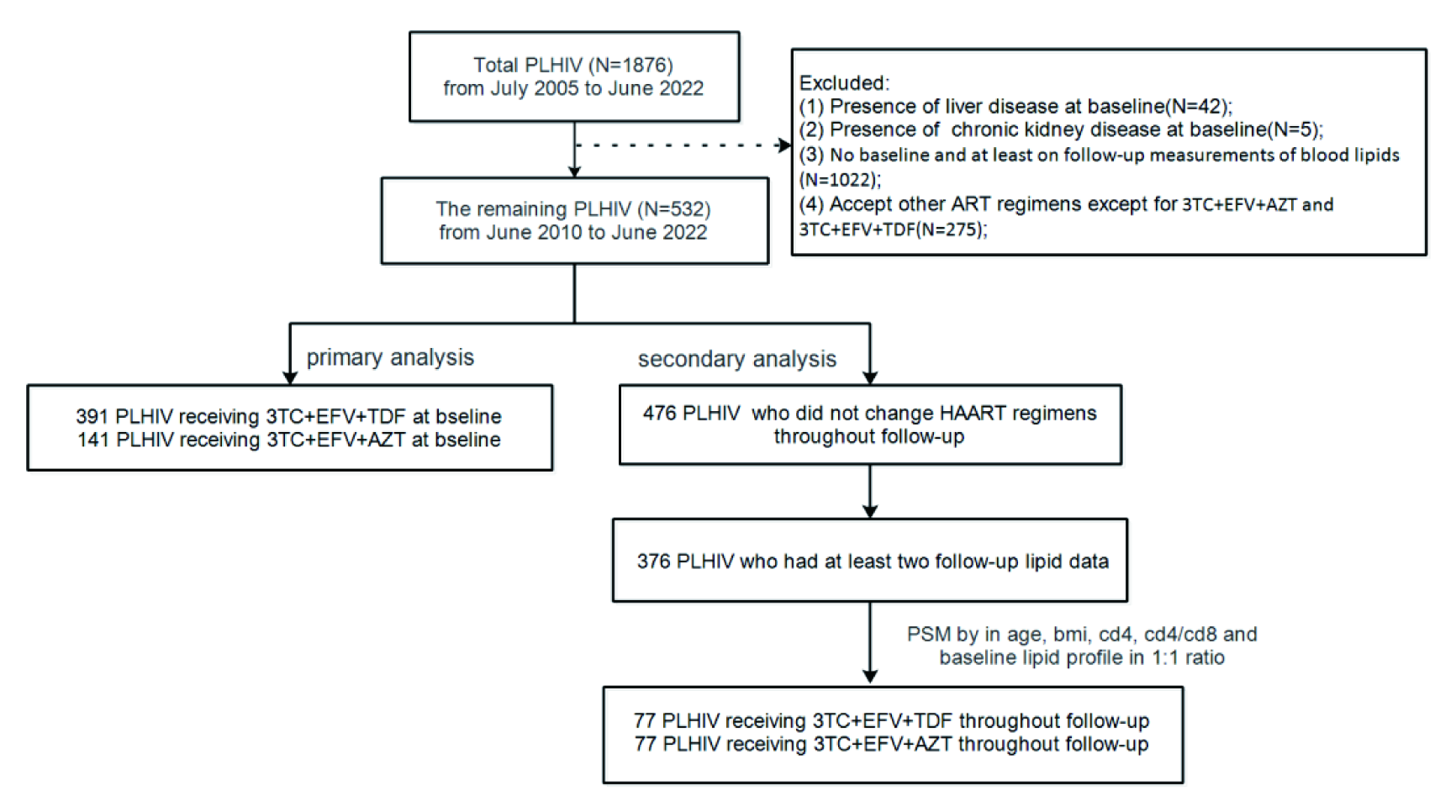

Supplement: S1 Fig — (TIF) [file pone.0305461.s001.tif]

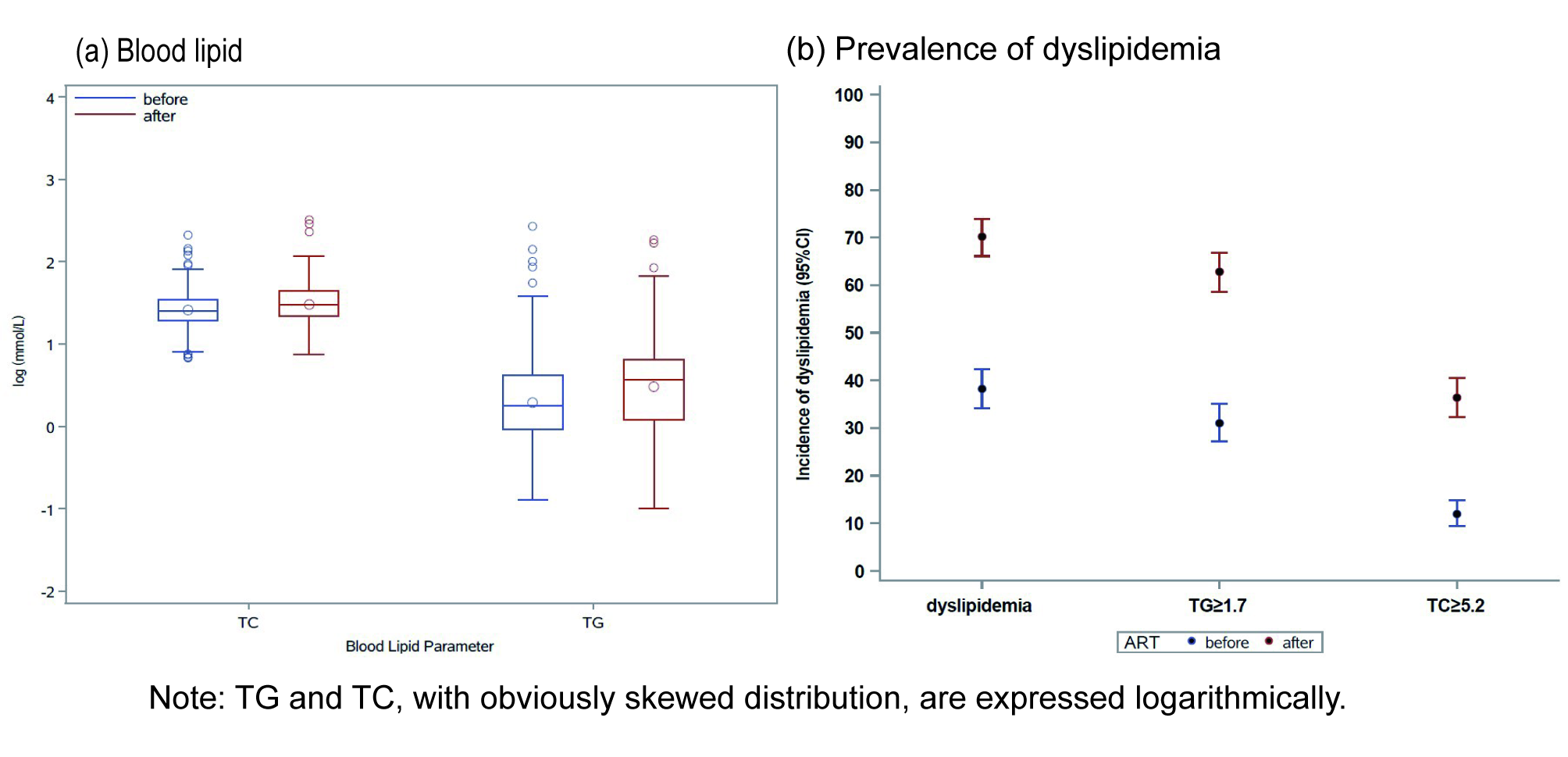

Supplement: S2 Fig — TG and TC with obviously skewed distribution, they are expressed logarithmically. (TIF) [file pone.0305461.s002.tif]

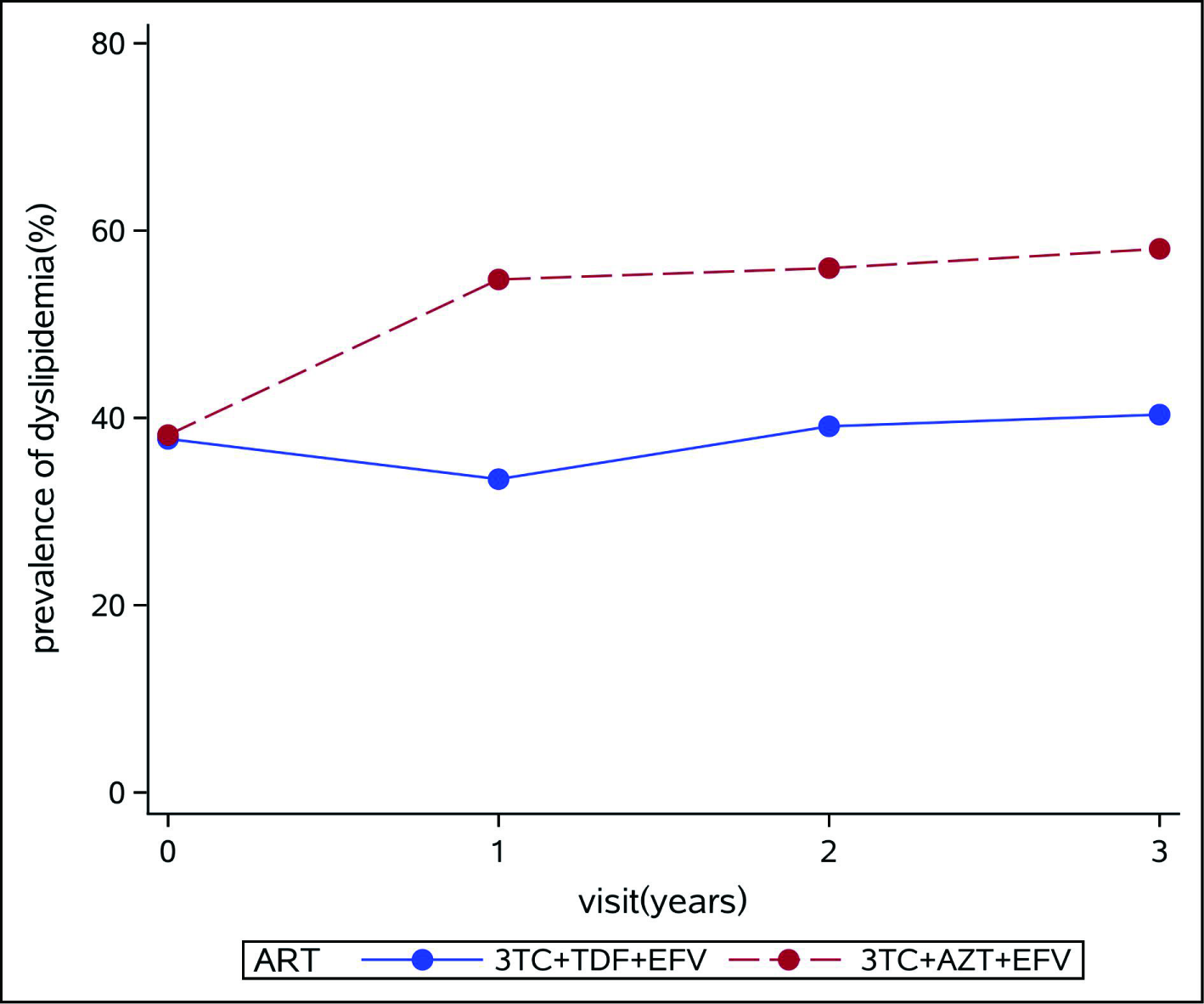

Supplement: S3 Fig — (TIF) [file pone.0305461.s003.tif]

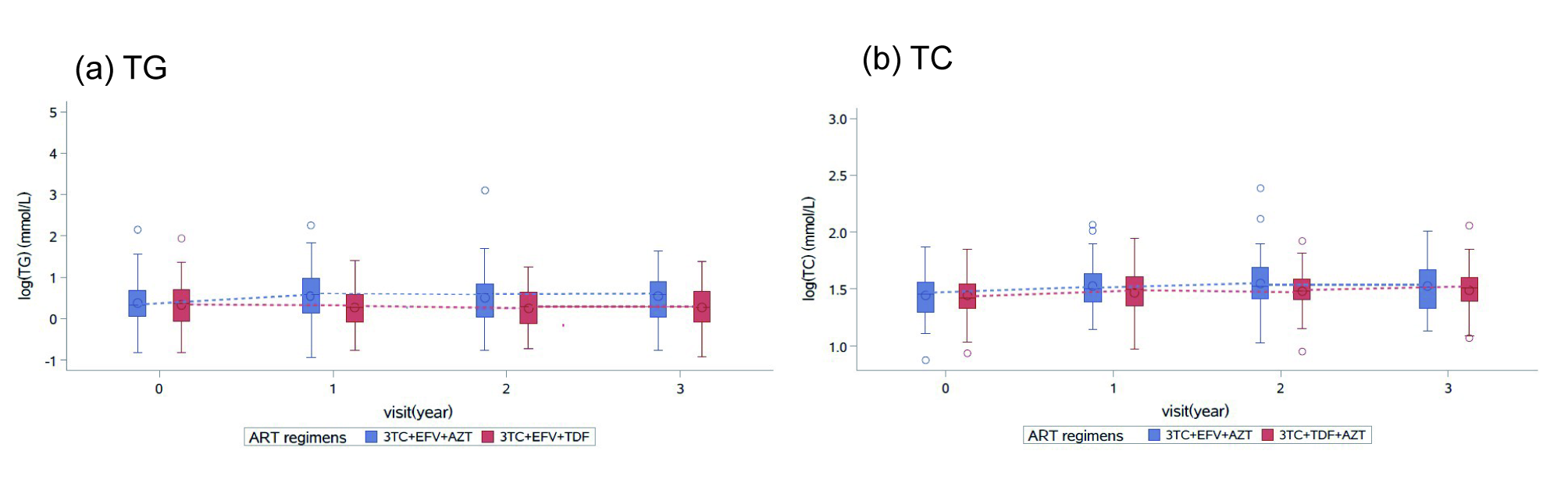

Supplement: S4 Fig — (TIF) [file pone.0305461.s004.tif]
