# Supplementary material for: The risk of dyslipidemia on PLHIV associated with different antiretroviral regimens in Huzhou
Source: PLoS One. 2024 Sep 20;19(9):e0305461. doi: 10.1371/journal.pone.0305461 (PMC11414983; doi:10.1371/journal.pone.0305461)
Supplement: S1 Table — Continuous variables was described as median (1st quartile, 3rd quartile) as its distribution was skewed and Mann-Whitney U test was applied to compare the difference between two groups; Categorical data were presented with number (%) and chi-square tests or Fisher’s exact test were used to compare the differences between pre- and post-imputations data. Abbreviations: CD4: CD4+ T-lymphocyte count; CD8: CD8+ T-lymphocyte count; WBC: White blood cell; ALT: Alanine aminotransferase; AST: Aspartate transaminase; TBIL: Total bilirubin; FPG: Fast plasma glucose. (DOCX) [file pone.0305461.s005.docx]

**S1 Table. Comparison of pre- and post-imputations by 5-fold multiple imputation**

| **Variables** | **Pre-imputations** | **Post-imputations** | **P-value** |
| --- | --- | --- | --- |
| Hight, m | 1.7(1.7,1.8) | 1.7(1.7,1.8) | 0.886 |
| Weight, kg | 64.0(57.0,71.0) | 64.0(58.0,70.0) | 0.831 |
| CD4, cells/L | 283.5(180.3,419.0) | 273.5(190.6,401.0) | 0.792 |
| CD8, cells/L | 769.0(549.1,1058.0) | 751.3(570.3,1008.0) | 0.599 |
| WBC, 10^9^/L | 5.3(4.3,6.5) | 5.3(4.3,6.5) | 0.784 |
| Platelet, 10^9^/L | 189.0(154.0,230.0) | 189.0(154.0,230.0) | 0.940 |
| Hemoglobin, g/L | 148.0(134.0,157.0) | 148.0(134.0,157.0) | 0.797 |
| ALT, U/L | 22.0(15.4,32.2) | 22.0(15.4,32.1) | 0.932 |
| AST, U/L | 22.0(18.8,28.1) | 22.1(19.0,28.0) | 0.498 |
| Creatinine, mmol/L | 72.8(64.3,82.3) | 72.8(64.4,82.3) | 0.881 |
| TBIL, mmol/L | 10.0(7.1,14.0) | 10.2(7.6,13.6) | 0.709 |
| FPG, mmol/L | 5.2(4.8,5.8) | 5.2(4.8,5.8) | 0.932 |

**Note:** Continuous variables are described as median (1^st^ quartile, 3^rd^ quartile) as their distribution ss skewed, and the Mann-Whitney U test is applied to compare the difference between two groups; categorical data are presented as number (%) and the chi-square tests or Fisher’s exact test are used to compare the differences between pre- and post-imputations data.

**Abbreviations:** CD4: CD4^+^ T-lymphocyte count; CD8: CD8^+^ T-lymphocyte count; WBC: White blood cell; ALT: Alanine aminotransferase; AST: Aspartate transaminase; TBIL: Total bilirubin; FPG: fasting plasma glucose.
