# Supplementary material for: The risk of dyslipidemia on PLHIV associated with different antiretroviral regimens in Huzhou
Source: PLoS One. 2024 Sep 20;19(9):e0305461. doi: 10.1371/journal.pone.0305461 (PMC11414983; doi:10.1371/journal.pone.0305461)
Supplement: S2 Table — Percentage of change = (prevalence of dyslipidemia after HAART- prevalence of dyslipidemia before HAART)/ prevalence of dyslipidemia before HAART. (DOCX) [file pone.0305461.s006.docx]

**S2 Table. Changes in the prevalence of dyslipidemia in patients receiving different ART regimens**

| **ART regimens** | **Outcomes** | **Before HAART (%)** | **After HAART (%)** | **Percentage of**  **change (%)** |
| --- | --- | --- | --- | --- |
| 3TC+ EFV+TDF  (N=376) | Dyslipidemia | 37.77 | 65.43 | 73.23 |
|  | TG≥1.7 | 29.79 | 57.18 | 91.94 |
|  | TC≥5.3 | 12.5 | 33.24 | 165.92 |
| 3TC+ EFV+AZT  (N=110) | Dyslipidemia | 38.18 | 78.18 | 104.77 |
|  | TG≥1.7 | 32.73 | 72.73 | 122.21 |
|  | TC≥5.3 | 11.82 | 43.64 | 269.20 |

**Note:** Percentage change = (prevalence of dyslipidemia after ART- prevalence of dyslipidemia before ART)/ prevalence of dyslipidemia before ART.
