# Supplementary material for: The risk of dyslipidemia on PLHIV associated with different antiretroviral regimens in Huzhou
Source: PLoS One. 2024 Sep 20;19(9):e0305461. doi: 10.1371/journal.pone.0305461 (PMC11414983; doi:10.1371/journal.pone.0305461)
Supplement: S3 Table — (DOCX) [file pone.0305461.s007.docx]

**S3 Table. Numbers of patients in the cohort at various follow-up points stratified by regimens**

| **Regimens** | **0 year** | **1 year** | **2 years** | **3 years** |
| --- | --- | --- | --- | --- |
| Total | 476 | 294 | 232 | 182 |
| 3TC+TDF+EFV | 376 | 245 | 202 | 166 |
| 3TC+AZT+EFV | 110 | 73 | 50 | 31 |
