# Supplementary material for: The risk of dyslipidemia on PLHIV associated with different antiretroviral regimens in Huzhou
Source: PLoS One. 2024 Sep 20;19(9):e0305461. doi: 10.1371/journal.pone.0305461 (PMC11414983; doi:10.1371/journal.pone.0305461)
Supplement: S4 Table — 77 pairs of PLHIV were included the gee model, in which one case (PLHIV receiving 3TC+EFV+AZT) was matched by age, BMI, cd4, cd8, TG and TC at baseline with one control (PLHIV receiving 3TC+EFV+TDF). (DOCX) [file pone.0305461.s008.docx]

**S4 Table. The gee model of effect of two ART regimens on the risk of dyslipidemia of PLHIV**

| **Outcomes** | **HAART regimens** | **OR (95%CI)** | **P-value** |
| --- | --- | --- | --- |
| Dyslipidemia | 3TC +EFV+TDF | Ref | Ref |
|  | 3TC +EFV+AZT | 1.46(0.88,2.43) | 0.148 |
| TG≥1.7 | 3TC +EFV+TDF | Ref | Ref |
|  | 3TC +EFV+AZT | 1.84(1.10,3.07) | 0.020 |
| TC≥5.3 | 3TC +EFV+TDF | Ref | Ref |
|  | 3TC +EFV+AZT | 1.52(0.78,2.96) | 0.219 |

**Note:** 77 pairs of PLHIV are included in the gee model, in which one case (PLHIV receving 3TC+EFV+AZT) is matched by age, BMI, CD4, CD8, TG and TC levels at baseline with one control (PLHIV receving 3TC+EFV+TDF).
