# Supplementary material for: The risk of dyslipidemia on PLHIV associated with different antiretroviral regimens in Huzhou
Source: PLoS One. 2024 Sep 20;19(9):e0305461. doi: 10.1371/journal.pone.0305461 (PMC11414983; doi:10.1371/journal.pone.0305461)
Supplement: Attachment — Submitted filename: Response to Reviewers.docx [file pone.0305461.s009.docx]

**Dear editor,**

**We hope that you are doing very well.**

**On behalf of all co-authors, I would like to thank you and the reviewers for your thoughtful review of our manuscript(Number: PONE-D-23-04082), and the opportunity you give us to revise the manuscript. We deeply appreciate these important comments and valuable suggestions offered by the reviewers and you, and have revised the manuscript, point by point, in response to the comments and suggestions.**

**Best,**

**Meihua Jin**

**Guangyun Mao, MD, PhD**

**COMMENTS TO THE AUTHOR:**

**Reviewer#1:**

***Question1.*** *I’m wondering about the data analysis of this study is logistic regression. I expected a time-to-event analysis or joint models since the data has a time component, repeated measurement of outcome variables (lipid profiles), and comparing the two HARRT regimens (3TC-EFV-TDF and 3TC-EFV-AZT). The epidemiological measures for cohort study with an open population are incidence density and measures of association again be relative risk or hazard ratio, but this paper reported prevalence rate and odds ratio for retrospective cohort study. I will be happy if the authors of this manuscript address these issues.*

**Response:** Thank you very much for this valuable comment. Our primary statistical method of choice is logistic regression model, rather than the Cox proportional hazards model. The reason for this is that our outcome of interest is dyslipidemia rather than death. Although various laboratory measurements, including triglyceride levels, were periodically assessed during the patient’s follow-up, we do not have access to the exact timing of when lipid abnormalities occurred. In other words, we are unable to obtain the true survival time of the patients. Therefore, our outcome is defined as the occurrence of elevated lipid profiles at any point during the follow-up period, which does not allow for Cox regression analysis. Additionally, to avoid wasting the repeated measurements taken during the follow-up, we employed the generalized estimating equation (GEE) model in the second analysis to evaluate the association between treatment regimen and lipid abnormalities among patients who did not change their treatment during the follow-up. Moreover, this study is a retrospective cohort study, and retrospective studies cannot calculate incidence rates. Since the relative risk (RR) is based on rates, the measure of association in this article is presented as odds ratio (OR) values.

**Reviewer#2:**

***Question1.*** *There are some typos in the lines 203-214 where dyslipidemia is written as* *"dyslipodemia", please correct it.*

**Response:** We are grateful for this kind reminder and sorry for this carelessness. We have completed the modification of “dyslipidemia”.

***Question2.*** *In line 32, method section of abstract, the authors wrote "from June 2010 to June 2022", while in all the other section that period seems to be from July 2005 to June 2022 (lines 80-81 and 91), please correct it*

**Response:** Thank you again for your kind reminder and we are sorry for our carelessness. We have corrected the error in line 32.

***Question3.*** *Data about the use of lowering triglycerides/cholesterol drugs are missing in the analysis. It would be interesting to know if the reduction in lipid profile was observed in people treated or not with those drugs. Could the authors provide this information about the participants, possibly making a sub-analysis that include this variable?*

**Response:** Many thanks for this important and interesting reminder. We completely agree with the reviewer. Unfortunately, there is no information in our data set on drug use other than antiviral regimens. We believe that if we obtain the relevant variables of lipid-lowering drugs, we may get some new results. In subsequent studies, we will recommend that CDC staff collect additional information about patients' drug use.
