# Supplementary material for: The risk of dyslipidemia on PLHIV associated with different antiretroviral regimens in Huzhou
Source: PLoS One. 2024 Sep 20;19(9):e0305461. doi: 10.1371/journal.pone.0305461 (PMC11414983; doi:10.1371/journal.pone.0305461)
Supplement: Attachment — Submitted filename: Response to Reviewers.docx [file pone.0305461.s010.docx]

**Dear editor,**

**We hope that you are doing very well.**

**On behalf of all co-authors, I would like to thank you and the reviewers for your thoughtful review of our manuscript(Number: PONE-D-23-04082), and the opportunity you give us to revise the manuscript. We deeply appreciate these important comments and valuable suggestions offered by the reviewers and you, and have revised the manuscript, point by point, in response to the comments and suggestions.**

**Best,**

**Meihua Jin**

**Guangyun Mao, MD, PhD**

**COMMENTS TO THE AUTHOR:**

**Reviewer#1:**

***Question.*** *It is clear that* *survival analysis can apply to any outcome with a time component, not just death. For example, patients with longer follow-up times have a higher chance of developing dyslipidaemia than patients with shorter follow-up times. So, the analysis should consider follow-up time accounting interval censoring; otherwise, the result will be misleading. In the GEE analysis, the authors* *assessed those with unchanged HAART regimen, here what if the change is because of high lipid profile, does it mean you introducing bias in the analysis. Related question: how did you manage patients dead or lost in the follow-up period and having one or more lipid measurements? Did you assess the computing risks?*

**Response:** Thank you very much for your valuable comments. It is well known that survival analysis can be utilized for outcomes with a time component, provided that we have a relatively accurate survival time, that is, between the start of the study and the occurrence of the event of interest. Death is just one example we provide, as we can usually ascertain the actual time of death. In clinical practice, the time of visit is often used as a proxy for the time of occurrence of a disease, despite the potential for measurement errors. However, the survival analysis method is robust to this situation in the data. But I would like to say the outcomes of our study differ from those mentioned above. Lipids were measured at each follow-up, and as long as a patient had dyslipidemia, we considered that the end event of interest had occurred. Furthermore, our lipid data are incomplete. It is likely that patients already had dyslipidemia at the first follow-up, but were missed due to a lack of HDL_C or LDL_C data. These individuals were either not identified until later follow-ups based on other lipid data, or remained undetected. Consequently, the outcome was more suitable for analysis as a binary event rather than as an event that included time. In summary, the case is specific. Generally, survival analysis methods should be selected for data containing time, but for this study, we believe that logistic regression analysis is more suitable.

In the GEE analysis, we assessed those with unchanged HAART regimen. First of all, we cannot deny that patients may have some adverse reactions during treatment, such as metabolic disorders, and change the HAART regimen, which will cause bias in the study results. However, we were unable to ascertain the specific reasons for patients' treatment regimen changes, thereby precluding an estimation of this bias. This constitutes a significant limitation of our study. Nonetheless, 89.5% (476/532) of patients maintained their treatment regimen, suggesting a minimal bias. Secondly, the GEE analysis necessitated repeated measurements, hence we retained patients with at least two follow-up records.

Considering the interference caused by confounding factors such as age and CD4, the relationship between HAART and dyslipidemia was analyzed after 1:1 matching. Ultimately, GEE analysis was performed on only 77 pairs of patients after receiving matching, and none of these 77 pairs disappeared or died during follow-up. What I would like to emphasize is that, despite the fact that only 154 subjects remain after pairing, GEE analysis serves as an auxiliary analysis supporting the main research findings in this study. This part of the results is also included in a supplementary document.Thank you again for your comments.

**Reviewer#3:**

***Question.*** *The authors have approached the subject in an easily understandable way. However, has the possibility of interference with other products taken by patients been taken into account? This could have been a factor influencing the advent of elevation of lipid profiles, can the authors give more details on this subject.*

**Response:** We are grateful for this kind reminder and we completely agree with your opinion. We must acknowledge that if patients take other medications, it may affect their blood lipid levels. Unfortunately, details of other drug use histories were missing from our database, which we will add to the limitations of the study.In subsequent studies, we will recommend that CDC staff collect additional information about patients' drug use.

**Reviewer#4:**

***Question1.*** *I understand that English might not be the first language of the authors, but they might want to seek professional services to improve the presentation and grammar. This can remarkably improve the readability and professional presentation of your manuscript.*

**Response:** Thank you for your kind reminder and we will continue to polish our article to improve its readability.

***Question2.*** *Research Question and Aims: The research question and specific aims of the study are not stated with sufficient clarity. Clearly articulating these elements is fundamental to guiding the reader through your research process and understanding the significance of your findings. I recommend revising the introduction to explicitly state the research question(s)/aims. For example, to estimate the prevalence of...., to assess the trend.... to evaluate the association of ART with dyslipedemia. When clearly stated readers expect to see results and discussion sections presented in alignment with the study aims. The current presentation is hard to follow.*

**Response:** We are grateful for this kind reminder. We have revised the research purpose of the introduction section to make it clearer and easier to understand.

***Question3.*** *Lines 269-272: The passage discussing the comparative adverse effects of different ART regimens on blood lipid metabolism seems more interpretative than is typical for a Results section. I recommend relocating or rephrasing this content to maintain a clear distinction between the presentation of findings (Results) and their interpretation (Discussion).*

**Response:** Many thanks for this important and interesting reminder. We have deleted and modified the statements in lines 269-272 to make the description more logical.

***Question4.*** *Including both unadjusted and adjusted regression analyses results for the covariates would offer a more comprehensive view of the data and the factors influencing dyslipidemia among PLHIV. Presenting the effect sizes of other potential determinants would also be beneficial in interpreting the results and understanding their broader implications. You could add it as supplementary data.*

**Response:** Many thanks for this kind reminder. However, we would like to say that this study is an association study, not an analysis of influencing factors. We had only one main independent variable, the HAART regimen, and the other variables in the multifactor model were corrected as confounders. And the effect sizes of HAART's effects on dyslipidemia, whether uncorrected or corrected, have been sorted out and placed in Table 3 of the paper. We do not think it is necessary to label the effect sizes of other confounding factors as this is not in line with the main idea of the article. Thank you again for your comments.
